# 6-Methyl-2,4-diphenyl­quinoline

**DOI:** 10.1107/S1600536808015651

**Published:** 2008-06-07

**Authors:** Xing Huo, Yanfen Xu, Xinyun Li, Xinfu Pan

**Affiliations:** a Department of Chemistry, State Key Laboratory of Applied Organic Chemistry, Lanzhou University, Lanzhou 730000, People’s Republic of China

## Abstract

The mol­ecules of the title compound, C_22_H_17_N, are linked by weak inter­actions, among which the most prominent are C—H⋯π inter­actions. The dihedral angles between the phenyl rings and the quinoline ring system are 43.3 (3) and 21.4 (3)°. The title product resulted from a three-component reaction of benzaldehyde, 1-ethynylbenzene and *p*-toluidine via C—H activation of 1-ethynylbenzene catalyzed by CuI in the ionic liquid 1-butyl-3-methyl­imidazolium hexa­fluoro­phosphate.

## Related literature

For related literature, see: Allen *et al.* (1987 ▶); Park & Alper (2005 ▶); Shi *et al.* (2004 ▶); Skraup (1880 ▶). 
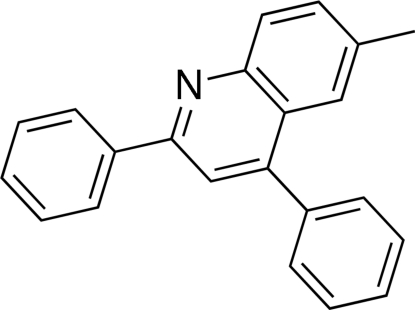


## Experimental

### 

#### Crystal data

C_22_H_17_N
*M* *_r_* = 295.37
Orthorhombic, 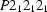

*a* = 7.766 (1) Å
*b* = 9.851 (1) Å
*c* = 20.756 (2) Å
*V* = 1588.0 (3) Å^3^
*Z* = 4
Mo *K*α radiation
μ = 0.07 mm^−1^
*T* = 294 (2) K
0.41 × 0.35 × 0.30 mm

#### Data collection

Bruker SMART CCD area-detector diffractometer
Absorption correction: multi-scan (*SADABS*; Sheldrick, 1996 ▶) *T*_min_ = 0.971, *T*_max_ = 0.979
8562 measured reflections
1720 independent reflections
1302 reflections with *I* > 2σ(*I*)
*R*_int_ = 0.051

#### Refinement

*R*[*F* ^2^ > 2σ(*F* ^2^)] = 0.043
*wR*(*F* ^2^) = 0.120
*S* = 1.04
1720 reflections
210 parameters
H-atom parameters constrained
Δρ_max_ = 0.17 e Å^−3^
Δρ_min_ = −0.14 e Å^−3^

### 

Data collection: *SMART* (Bruker, 1998 ▶); cell refinement: *SMART*; data reduction: *SAINT* (Bruker, 1998 ▶); program(s) used to solve structure: *SHELXS97* (Sheldrick, 2008 ▶); program(s) used to refine structure: *SHELXL97* (Sheldrick, 2008 ▶); molecular graphics: *SHELXTL* (Sheldrick, 2008 ▶); software used to prepare material for publication: *SHELXTL*.

## Supplementary Material

Crystal structure: contains datablocks global, I. DOI: 10.1107/S1600536808015651/fb2095sup1.cif

Structure factors: contains datablocks I. DOI: 10.1107/S1600536808015651/fb2095Isup2.hkl

Additional supplementary materials:  crystallographic information; 3D view; checkCIF report

## Figures and Tables

**Table 1:** Hydrogen-bond geometry (Å, °) *Cg*2 and *Cg*3 are the centroids of the C1–C6 and C14–C19 rings, respectively .

*D*—H⋯*A*	*D*—H	H⋯*A*	*D*⋯*A*	*D*—H⋯*A*
C6—H6⋯*Cg*3^i^	0.93	2.75	3.551 (3)	145
C11—H11⋯*Cg*2^i^	0.93	2.92	3.726 (3)	146

Symmetry code: (i) 
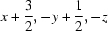
.

**Fig. 1.**
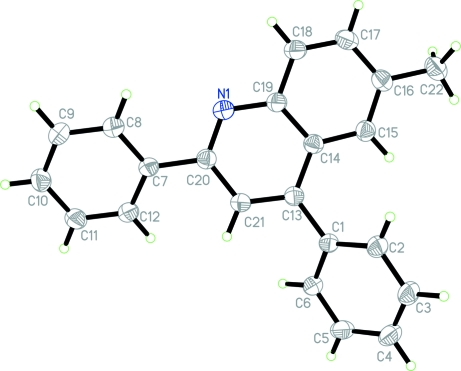
The molecular structure with the atom-numbering scheme. The displacement ellipsoids are drawn at the 30% probability level.

**Fig. 2.**
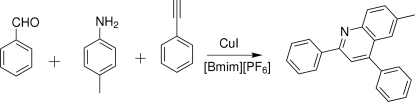
The formation of the title compound.

## supplementary crystallographic information

### Comment

Quinolines and their derivatives are very important in medical chemistry because of their extensive occurrence in natural products. Also, quinolines possess a wide spectrum of biological activities. The classic method of quinoline synthesis is Skraup's procedure (Skraup, 1880). The synthesis of the title compound follows a study of transition-metal catalyzed multi-component reactions which is a powerful synthetic tool to access complex structures from simple precursors by a one-pot procedure (Shi *et al.*, 2004; Park & Alper, 2005).

In the title compound, all the bond lengths are  normal  (Allen *et al.*, 1987). The  angle between both phenyl rings in the structure is 34.6 (3) °. The dihedral angle between the  phenyl ring C1—C6 and the  ring C14—C21/N1 is 43.3 (3) °. The dihedral angle between the C7—C12 phenyl ring and the C14—C21/N1 ring is 21.4 (3) °.

### Experimental

The *p*-toluidine (1.5 mmol) and benzaldehyde(1.5 mmol) were taken along with a catalytic quantity of CuI (0.45 mmol) in ionic liquid 1-butyl-3-methylimidazolium hexafluorophosphate (5 ml) (Scheme 2). The resulting mixture was stirred at 298 K for 15 minutes. At this stage 1-ethynylbenzene (1 mmol) was quickly poured into the reaction mixture and the temperature was raised to 404 K, kept at this temperature for 6 hours, then cooled to room temperature. The product was extracted from the reaction mixture by addition of diethyl ether. (It was possible to recover ionic liquid layer and to use it again without any pretreatment.) The combined organic layer was concentrated and the desired product was isolated by silica gel column chromatography(petrol/EtOAc, 20:1). Colourless sheet crystals were recrystallized from the deuterated chloroform CDCl3 by evaporation in the course of several days. Their average size was 2.5-3 mm.

### Refinement

Though all the H atoms could be located in the difference electron-density maps, they were placed into the idealized positions and constrained to ride on their parent atoms. The constrained distances: C—H = 0.93 or 0.96 Å for the aryl or the methyl hydrogens, respectively. The hydrogens' isotropic displacement parameters : *U*_iso_(H) = 1.2*U*_eq_(C) or 1.5*U*_eq_(C) for aryl or methyl hydrogens, respectively. In the absence of significant anomalous scattering effects 1413 Friedel pairs have been merged.

### Crystal data

**Table:**

C_22_H_17_N	*F*_000_ = 624
*M**_r_* = 295.37	*D*_x_ = 1.235 Mg m^−^^3^
Orthorhombic, *P*2_1_2_1_2_1_	Mo *K*α radiation λ = 0.71073 Å
Hall symbol: P2ac2ab	Cell parameters from 803 reflections
*a* = 7.766 (1) Å	θ = 2.3–24.5º
*b* = 9.851 (1) Å	µ = 0.07 mm^−^^1^
*c* = 20.756 (2) Å	*T* = 294 (2) K
*V* = 1588.0 (3) Å^3^	Block, colourless
*Z* = 4	0.41 × 0.35 × 0.30 mm

### Data collection

**Table:**

Bruker SMART CCD area-detector diffractometer	1720 independent reflections
Radiation source: fine-focus sealed tube	1302 reflections with *I* > 2σ(*I*)
Monochromator: graphite	*R*_int_ = 0.052
*T* = 294(2) K	θ_max_ = 25.5º
ω scans	θ_min_ = 2.0º
Absorption correction: multi-scan(SADABS; Sheldrick, 1996)	*h* = −8→9
*T*_min_ = 0.971, *T*_max_ = 0.979	*k* = −11→11
8562 measured reflections	*l* = −25→22

### Refinement

**Table:**

Refinement on *F*^2^	Secondary atom site location: difference Fourier map
Least-squares matrix: full	Hydrogen site location: difference Fourier map
*R*[*F*^2^ > 2σ(*F*^2^)] = 0.043	H-atom parameters constrained
*wR*(*F*^2^) = 0.120	*w* = 1/[σ^2^(*F*_o_^2^) + (0.0684*P*)^2^] where *P* = (*F*_o_^2^ + 2*F*_c_^2^)/3
*S* = 1.04	(Δ/σ)_max_ < 0.001
1720 reflections	Δρ_max_ = 0.17 e Å^−^^3^
210 parameters	Δρ_min_ = −0.14 e Å^−^^3^
67 constraints	Extinction correction: SHELXL97 (Sheldrick, 2008)
Primary atom site location: structure-invariant direct methods	Extinction coefficient: 0.009 (2)

### Special details

**Table:**

Geometry. All e.s.d.'s (except the e.s.d. in the dihedral angle between two l.s. planes) are estimated using the full covariance matrix. The cell e.s.d.'s are taken into account individually in the estimation of e.s.d.'s in distances, angles and torsion angles; correlations between e.s.d.'s in cell parameters are only used when they are defined by crystal symmetry. An approximate (isotropic) treatment of cell e.s.d.'s is used for estimating e.s.d.'s involving l.s. planes.
Refinement. Refinement of *F*^2^ against ALL reflections. The weighted *R*-factor *wR* and goodness of fit *S* are based on *F*^2^, conventional *R*-factors *R* are based on *F*, with *F* set to zero for negative *F*^2^. The threshold expression of *F*^2^ > σ(*F*^2^) is used only for calculating *R*-factors(gt) *etc*. and is not relevant to the choice of reflections for refinement. *R*-factors based on *F*^2^ are statistically about twice as large as those based on *F*, and *R*- factors based on ALL data will be even larger.

### Fractional atomic coordinates and isotropic or equivalent isotropic displacement parameters (Å^2^)

**Table:**

	*x*	*y*	*z*	*U*_iso_*/*U*_eq_	
N1	0.8369 (3)	0.4866 (3)	0.14992 (11)	0.0493 (6)	
C1	0.6966 (4)	0.5970 (3)	0.34443 (12)	0.0453 (7)	
C2	0.7800 (4)	0.5422 (3)	0.39763 (13)	0.0538 (8)	
H2	0.8605	0.4733	0.3920	0.065*	
C3	0.7440 (5)	0.5893 (4)	0.45891 (14)	0.0640 (9)	
H3	0.7997	0.5513	0.4942	0.077*	
C4	0.6258 (5)	0.6926 (4)	0.46804 (14)	0.0678 (10)	
H4	0.6016	0.7238	0.5093	0.081*	
C5	0.5439 (5)	0.7489 (3)	0.41549 (13)	0.0621 (9)	
H5	0.4649	0.8188	0.4214	0.074*	
C6	0.5788 (4)	0.7021 (3)	0.35440 (13)	0.0493 (7)	
H6	0.5232	0.7409	0.3193	0.059*	
C7	0.8147 (4)	0.7190 (3)	0.11564 (12)	0.0448 (7)	
C8	0.9118 (4)	0.6959 (3)	0.06060 (13)	0.0562 (8)	
H8	0.9718	0.6147	0.0562	0.067*	
C9	0.9206 (5)	0.7918 (3)	0.01228 (14)	0.0647 (9)	
H9	0.9870	0.7750	−0.0242	0.078*	
C10	0.8318 (5)	0.9124 (3)	0.01757 (15)	0.0638 (9)	
H10	0.8375	0.9767	−0.0152	0.077*	
C11	0.7347 (4)	0.9371 (3)	0.07175 (14)	0.0592 (9)	
H11	0.6745	1.0182	0.0758	0.071*	
C12	0.7268 (4)	0.8410 (3)	0.12009 (13)	0.0524 (8)	
H12	0.6608	0.8586	0.1565	0.063*	
C13	0.7370 (3)	0.5530 (3)	0.27752 (13)	0.0434 (7)	
C14	0.7596 (3)	0.4139 (3)	0.25973 (13)	0.0448 (7)	
C15	0.7277 (4)	0.3018 (3)	0.30025 (13)	0.0492 (7)	
H15	0.6855	0.3175	0.3415	0.059*	
C16	0.7564 (4)	0.1704 (3)	0.28110 (15)	0.0522 (8)	
C17	0.8282 (4)	0.1481 (3)	0.22001 (14)	0.0559 (8)	
H17	0.8570	0.0602	0.2077	0.067*	
C18	0.8566 (4)	0.2523 (3)	0.17834 (14)	0.0545 (8)	
H18	0.9014	0.2342	0.1377	0.065*	
C19	0.8189 (4)	0.3880 (3)	0.19587 (13)	0.0454 (7)	
C20	0.8015 (4)	0.6132 (3)	0.16636 (13)	0.0447 (7)	
C21	0.7571 (4)	0.6497 (3)	0.23033 (12)	0.0472 (7)	
H21	0.7413	0.7407	0.2406	0.057*	
C22	0.7120 (5)	0.0522 (3)	0.32401 (16)	0.0677 (10)	
H22A	0.6644	0.0853	0.3637	0.102*	
H22B	0.8141	0.0007	0.3329	0.102*	
H22C	0.6291	−0.0046	0.3028	0.102*	

### Atomic displacement parameters (Å^2^)

**Table:**

	*U*^11^	*U*^22^	*U*^33^	*U*^12^	*U*^13^	*U*^23^
N1	0.0537 (14)	0.0477 (15)	0.0465 (14)	0.0033 (13)	0.0026 (11)	−0.0044 (11)
C1	0.0542 (16)	0.0405 (15)	0.0412 (15)	−0.0047 (14)	−0.0011 (13)	−0.0024 (13)
C2	0.0665 (19)	0.0507 (18)	0.0444 (16)	−0.0062 (16)	−0.0071 (14)	−0.0017 (13)
C3	0.086 (2)	0.062 (2)	0.0436 (16)	−0.012 (2)	−0.0136 (16)	−0.0016 (16)
C4	0.094 (3)	0.068 (2)	0.0423 (17)	−0.014 (2)	0.0028 (17)	−0.0100 (16)
C5	0.076 (2)	0.0515 (19)	0.0586 (18)	−0.0029 (18)	0.0090 (17)	−0.0136 (17)
C6	0.0576 (17)	0.0474 (17)	0.0429 (15)	−0.0015 (16)	0.0037 (13)	−0.0029 (14)
C7	0.0497 (16)	0.0444 (17)	0.0404 (14)	0.0003 (14)	−0.0010 (13)	−0.0034 (13)
C8	0.0662 (19)	0.0513 (19)	0.0512 (17)	0.0081 (17)	0.0083 (15)	−0.0005 (15)
C9	0.084 (2)	0.064 (2)	0.0465 (17)	0.002 (2)	0.0151 (16)	0.0002 (17)
C10	0.092 (2)	0.052 (2)	0.0480 (18)	−0.004 (2)	−0.0054 (18)	0.0059 (16)
C11	0.075 (2)	0.0471 (18)	0.0557 (18)	0.0086 (18)	−0.0039 (16)	0.0001 (15)
C12	0.0601 (17)	0.0519 (18)	0.0451 (15)	0.0038 (16)	0.0013 (14)	−0.0036 (15)
C13	0.0448 (16)	0.0426 (16)	0.0428 (14)	−0.0009 (14)	−0.0007 (13)	−0.0007 (13)
C14	0.0447 (16)	0.0441 (16)	0.0455 (15)	0.0025 (14)	−0.0010 (12)	−0.0017 (13)
C15	0.0521 (17)	0.0498 (18)	0.0458 (15)	−0.0017 (15)	−0.0033 (13)	0.0019 (13)
C16	0.0533 (18)	0.0454 (18)	0.0579 (17)	0.0014 (15)	−0.0086 (15)	0.0009 (15)
C17	0.0617 (18)	0.0445 (18)	0.0616 (19)	0.0077 (16)	−0.0076 (16)	−0.0066 (16)
C18	0.0588 (18)	0.0524 (19)	0.0522 (18)	0.0091 (17)	0.0026 (14)	−0.0061 (17)
C19	0.0471 (15)	0.0438 (17)	0.0453 (15)	0.0023 (14)	0.0005 (13)	−0.0021 (13)
C20	0.0452 (15)	0.0453 (17)	0.0437 (15)	−0.0013 (14)	−0.0009 (12)	−0.0006 (13)
C21	0.0549 (17)	0.0417 (16)	0.0451 (15)	0.0032 (14)	0.0004 (14)	−0.0040 (14)
C22	0.079 (2)	0.0488 (19)	0.076 (2)	−0.0005 (18)	−0.0066 (19)	0.0077 (18)

### Geometric parameters (Å, °)

**Table:**

N1—C20	1.322 (4)	C10—H10	0.9300
N1—C19	1.369 (3)	C11—C12	1.381 (4)
C1—C2	1.390 (4)	C11—H11	0.9300
C1—C6	1.396 (4)	C12—H12	0.9300
C1—C13	1.489 (4)	C13—C21	1.375 (4)
C2—C3	1.383 (4)	C13—C14	1.430 (4)
C2—H2	0.9300	C14—C15	1.410 (4)
C3—C4	1.384 (5)	C14—C19	1.426 (3)
C3—H3	0.9300	C15—C16	1.373 (4)
C4—C5	1.379 (4)	C15—H15	0.9300
C4—H4	0.9300	C16—C17	1.403 (4)
C5—C6	1.376 (3)	C16—C22	1.506 (4)
C5—H5	0.9300	C17—C18	1.361 (4)
C6—H6	0.9300	C17—H17	0.9300
C7—C12	1.385 (4)	C18—C19	1.416 (4)
C7—C8	1.388 (4)	C18—H18	0.9300
C7—C20	1.485 (4)	C20—C21	1.418 (4)
C8—C9	1.380 (4)	C21—H21	0.9300
C8—H8	0.9300	C22—H22A	0.9600
C9—C10	1.378 (5)	C22—H22B	0.9600
C9—H9	0.9300	C22—H22C	0.9600
C10—C11	1.376 (4)		
			
C20—N1—C19	118.0 (2)	C11—C12—H12	119.2
C2—C1—C6	118.4 (3)	C7—C12—H12	119.2
C2—C1—C13	122.0 (3)	C21—C13—C14	117.8 (2)
C6—C1—C13	119.5 (2)	C21—C13—C1	119.1 (3)
C3—C2—C1	120.4 (3)	C14—C13—C1	123.1 (2)
C3—C2—H2	119.8	C15—C14—C19	118.1 (3)
C1—C2—H2	119.8	C15—C14—C13	125.1 (2)
C2—C3—C4	120.5 (3)	C19—C14—C13	116.8 (3)
C2—C3—H3	119.8	C16—C15—C14	122.5 (3)
C4—C3—H3	119.8	C16—C15—H15	118.8
C5—C4—C3	119.6 (3)	C14—C15—H15	118.8
C5—C4—H4	120.2	C15—C16—C17	118.3 (3)
C3—C4—H4	120.2	C15—C16—C22	121.4 (3)
C6—C5—C4	120.2 (3)	C17—C16—C22	120.3 (3)
C6—C5—H5	119.9	C18—C17—C16	121.4 (3)
C4—C5—H5	119.9	C18—C17—H17	119.3
C5—C6—C1	120.9 (3)	C16—C17—H17	119.3
C5—C6—H6	119.5	C17—C18—C19	121.0 (3)
C1—C6—H6	119.5	C17—C18—H18	119.5
C12—C7—C8	117.7 (3)	C19—C18—H18	119.5
C12—C7—C20	121.8 (2)	N1—C19—C18	118.0 (2)
C8—C7—C20	120.4 (3)	N1—C19—C14	123.6 (3)
C9—C8—C7	120.9 (3)	C18—C19—C14	118.3 (3)
C9—C8—H8	119.6	N1—C20—C21	122.1 (3)
C7—C8—H8	119.6	N1—C20—C7	117.7 (2)
C10—C9—C8	120.5 (3)	C21—C20—C7	120.2 (3)
C10—C9—H9	119.7	C13—C21—C20	121.3 (3)
C8—C9—H9	119.7	C13—C21—H21	119.4
C11—C10—C9	119.4 (3)	C20—C21—H21	119.4
C11—C10—H10	120.3	C16—C22—H22A	109.5
C9—C10—H10	120.3	C16—C22—H22B	109.5
C10—C11—C12	119.8 (3)	H22A—C22—H22B	109.5
C10—C11—H11	120.1	C16—C22—H22C	109.5
C12—C11—H11	120.1	H22A—C22—H22C	109.5
C11—C12—C7	121.6 (3)	H22B—C22—H22C	109.5
			
C6—C1—C2—C3	1.2 (4)	C13—C14—C15—C16	−178.1 (3)
C13—C1—C2—C3	177.5 (3)	C14—C15—C16—C17	3.2 (4)
C1—C2—C3—C4	−0.6 (5)	C14—C15—C16—C22	−176.6 (3)
C2—C3—C4—C5	−0.2 (5)	C15—C16—C17—C18	−5.1 (5)
C3—C4—C5—C6	0.4 (5)	C22—C16—C17—C18	174.6 (3)
C4—C5—C6—C1	0.2 (5)	C16—C17—C18—C19	1.8 (5)
C2—C1—C6—C5	−1.0 (4)	C20—N1—C19—C18	179.9 (3)
C13—C1—C6—C5	−177.3 (3)	C20—N1—C19—C14	1.8 (4)
C12—C7—C8—C9	0.3 (4)	C17—C18—C19—N1	−174.7 (3)
C20—C7—C8—C9	178.0 (3)	C17—C18—C19—C14	3.5 (5)
C7—C8—C9—C10	−0.5 (5)	C15—C14—C19—N1	172.8 (3)
C8—C9—C10—C11	0.4 (5)	C13—C14—C19—N1	−7.1 (4)
C9—C10—C11—C12	−0.1 (5)	C15—C14—C19—C18	−5.3 (4)
C10—C11—C12—C7	0.0 (5)	C13—C14—C19—C18	174.8 (3)
C8—C7—C12—C11	−0.1 (4)	C19—N1—C20—C21	4.1 (4)
C20—C7—C12—C11	−177.7 (3)	C19—N1—C20—C7	−177.7 (2)
C2—C1—C13—C21	−135.1 (3)	C12—C7—C20—N1	157.1 (3)
C6—C1—C13—C21	41.1 (4)	C8—C7—C20—N1	−20.5 (4)
C2—C1—C13—C14	43.6 (4)	C12—C7—C20—C21	−24.7 (4)
C6—C1—C13—C14	−140.2 (3)	C8—C7—C20—C21	157.8 (3)
C21—C13—C14—C15	−173.5 (3)	C14—C13—C21—C20	−1.0 (4)
C1—C13—C14—C15	7.8 (4)	C1—C13—C21—C20	177.8 (2)
C21—C13—C14—C19	6.3 (4)	N1—C20—C21—C13	−4.6 (4)
C1—C13—C14—C19	−172.4 (3)	C7—C20—C21—C13	177.2 (3)
C19—C14—C15—C16	2.0 (4)		

### Hydrogen-bond geometry (Å, °)

**Table:**

*D*—H···*A*	*D*—H	H···*A*	*D*···*A*	*D*—H···*A*
C6—H6···Cg3^i^	0.93	2.75	3.551 (3)	145
C11—H11···Cg2^i^	0.93	2.92	3.726 (3)	146

Symmetry codes: (i) *x*+3/2, −*y*+1/2, −*z*.
